# “I don’t have the heart”: a qualitative study of barriers to and facilitators of physical activity for people with coronary heart disease and depressive symptoms

**DOI:** 10.1186/1479-5868-9-140

**Published:** 2012-11-30

**Authors:** Michelle C Rogerson, Barbara M Murphy, Stephen Bird, Tony Morris

**Affiliations:** 1 Heart Research Centre, The Royal Melbourne Hospital, PO Box 2137, Post Office, Melbourne, VIC, 3050, Australia; 2 Department of Psychiatry, The University of Melbourne, Grattan St, Parkville, VIC, 3052, Australia; 3 Health Innovations Research Institute and School of Medical Sciences, RMIT University, PO Box 71, Bundoora, VIC, 3083, Australia; 4 School of Sport and Exercise Science & Institute of Sport, Exercise and Active Living (ISEAL), Victoria University, PO Box 14428, Melbourne, VIC, 8001, Australia

**Keywords:** Coronary heart disease, Depressive symptoms, Physical activity, Barriers, Facilitators, Qualitative

## Abstract

**Background:**

Physical activity has been shown to reduce depression in people with coronary heart disease (CHD), however many people with CHD do not engage in sufficient levels of physical activity to reap its positive effects. People with depression and CHD are at particular risk of non-adherence to physical activity. Little is known about the barriers to and facilitators of physical activity for people with CHD and depressive symptoms. Using qualitative interviews, the aim of this study was to explore the barriers to and facilitators of physical activity for cardiac patients with depressive symptoms.

**Methods:**

Fifteen participants with CHD and depressive symptoms (assessed using the Cardiac Depression Scale) participated in in-depth semi-structured interviews. The interviews were focussed on investigating participants’ experiences of physical activity since their cardiac event. Interviews were content analysed to determine major themes.

**Results:**

Participants identified a number of barriers to and facilitators of physical activity. Barriers included having negative perceptions towards health and life changes as a result of the cardiac event, having low mood and low motivation to exercise, feeling physically restricted towards or fearful of exercise, lacking knowledge regarding exercise and perceiving external barriers. Facilitators included having a reason for exercising, being able to identify the psychological benefits of exercise, having positive social support and using psychological strategies. ‘Inactive’ participants reported more barriers and fewer facilitators than did ‘active’ participants.

**Conclusions:**

The barriers reported in this study were highly salient for a number of participants. Health professionals and researchers can use this information to assist people with CHD and depressive symptoms to identify and possibly overcome barriers to physical activity. Relevant barriers and facilitators could be taken into account to increase their effectiveness when designing interventions to encourage physical activity maintenance in this population.

## Background

Depression is common among people with coronary heart disease (CHD). Prevalence rates of major depression are between 17% and 27% in the months soon after an acute event, and many more people experience depressive symptoms [1]. A high prevalence of depression is still evident up to 12 months after a cardiac event [2]. As well as being a risk factor for the development of CHD [3], depression is associated with poor outcomes following a cardiac event, including increased cardiac-related mortality and morbidity [4].

Physical activity (PA) has been shown to reduce depression in people with CHD [5], being as effective as anti-depression medication for managing mild to moderate depression [6]. However, many people with CHD do not engage in sufficient levels of PA to gain its positive effects [7]. Depression can exacerbate non-adherence to PA for people with CHD [8].

Most studies that have investigated maintenance of PA in cardiac populations have used quantitative approaches. Quantitative studies are very informative, providing an overall analysis of relationships between variables and of the extent of the particular phenomena. However, qualitative methods can be useful for gaining a richer, more personal understanding of people’s specific experiences [9,10]. Over the past decade, a small number of qualitative studies have explored the reasons why people adhere to cardiac rehabilitation (CR) programs e.g. [11,12]. However, very little qualitative research has been undertaken to investigate PA experiences of people after participation in CR. One exception is the study by Fleury and colleagues [13], investigating barriers to PA for this population. They found that intrapersonal barriers such as perceived physical condition, a lack of motivation and interest, and competing demands influenced cessation of PA after CR. It is crucial to learn more about the barriers to and facilitators of PA following CR program attendance, given that research has demonstrated that attrition from post-CR exercise is high and increases over time [14].

Typically, individuals with depression are less likely to maintain an exercise program than their non-depressed counterparts in both cardiac [8] and non-cardiac [15,16] populations. Faulkner and Biddle [17] provided a rare insight into the in-depth experiences of PA of three individuals with clinical depression and highlighted the negative effects that depression had on their ability to maintain PA programs. The study illuminated the importance of individualised motives in maintaining PA and identified the symptom of lethargy as a barrier to PA for the three participants.

To our knowledge, no qualitative studies have investigated the experiences of PA for people with *both* depressive symptoms and CHD, despite the evidence suggesting the benefits of and barriers to PA for this population. Given the negative effects of depression on PA in people with CHD [8], it is possible that experiences of PA for CHD patients with depression may differ from those experienced by CHD patients who are not depressed. Therefore, using qualitative interviews, the aim of this study was to explore the barriers to and facilitators of PA for cardiac patients with depressive symptoms. In particular, it was predicted that inactive participants would report more barriers to, and fewer facilitators of PA than would active participants.

## Methods

### Participants

Fifteen participants (12 males; 3 females) who ranged in age from 47 – 75 years (*M* = 63.6 years) were recruited into the study. All participants had been hospitalised for a cardiac event within the past 12 months triggering referral to a CR program through Western Health in Victoria, Australia. Participants in the present study had all taken part in a questionnaire-based study (N = 102) with authors M.R, T.M and S.B, at which time they indicated their willingness to be contacted about further research. As part of the previous study, participants had completed the Cardiac Depression Scale (CDS) [18]. The inclusion criterion for the current study was that participants had scored between 100 and 125 on the CDS, which is considered to be indicative of ‘more severe’ depressive symptoms [19]. Out of the 65 participants who had expressed interest in further research, 17 participants were eligible according to their score on the CDS. Another inclusion criterion was fluency in English: two prospective participants were excluded on this basis. Participants were from an Australian (n = 10) or European background (n = 5). Almost half of the participants had less than a Year 10 education (n = 7). Six participants were regularly physically active according to the National Heart Foundation of Australia’s PA recommendations for people with cardiovascular disease [20] and nine participants were not regularly physically active. They were classified as ‘active’ and ‘inactive’ respectively. Characteristics of study participants are shown in Table 1. These characteristics were similar to the larger study population as well as to other studies of cardiac patients in Australia [21].

**Table 1:** Characteristics of study participants

	**n**	**%**
Gender		
Male	12	80
Female	3	20
Background		
Non-English speaking background	3	20
English-speaking background	12	80
Paid employment		
Yes	4	27
No	11	73
Education		
< Year 10	7	47
Year 11 or 12	5	33
Post-Year 12 (including trade)	3	20
Time since cardiac event		
<6 months	6	40
6-12 months	9	60
Event type		
Acute myocardial infarction	5	33
Percutaneous coronary intervention	5	33
Coronary artery bypass graft	3	20
Repaired/replaced valve	2	14
Sufficiently active*		
Yes	6	40
No	9	60

*Note*. *sufficiently active according to National Heart Foundation of Australia’s PA recommendations (i.e., participating in at least 30 min of moderate intensity PA on most, if not all, days of the week).

### Measures

#### Cardiac depression scale

The CDS [18] comprises 26 items, and measures depressed mood/depressive symptoms in people who have experienced a cardiac episode. Shi et al. [22] validated the CDS against the DSM-IV and reported a sensitivity of 94% and specificity of 77%. The internal reliability (Cronbach’s alpha = 0.90) is high [18,23], and concurrent validation against the Beck Depression Inventory (*r* = 0.79) and the Hospital Anxiety and Depression Scale (*r* = 0.77) show strong correlations [23]. The present study focuses on people with depressive symptoms, which the CDS measures, and not a Major Depressive Episode, which can only be assessed via a clinical interview.

### Procedure

After gaining approval from Victoria University Human Research Ethics Committee and Melbourne Health’s Mental Health Research and Ethics Committee, eligible participants were invited to take part in an in-depth, semi structured interview. The CDS was completed up to four weeks prior to the interview as part of the participants’ involvement in the previous questionnaire-based study. CDS scores were used to determine those participants who were experiencing depressive symptoms. The initial questions, designed to build rapport with participants, included a discussion of previous occupations, family and past experience with PA. The interviews then focussed on investigating participants’ experiences of PA since their cardiac event. Open-ended questions were used to ascertain what participants believed to be the major barriers and facilitators that affected their participation in PA. Elaboration and clarification probes [9] were used to explore issues in more depth. Interviews ranged in duration from 60–90 min.

Interviews were held at a convenient location (either the participant’s home or Victoria University) and time for each participant. The first author (M.R) conducted all interviews. The interviews were audio-taped with participants’ permission then transcribed verbatim. At the conclusion of the interview, participants were debriefed by the interviewer and thanked for their involvement. Confidentiality was maintained at all times when dealing with the transcripts to ensure individual participants could not be identified through quotations and descriptions.

### Analysis

After transcription, the interviews were analysed using procedures recommended by Patton [9] and successfully adapted to exercise and sport by Scanlan, Ravizza and Stein [24] and Gould, Jackson and Finch [25]. An inductive approach to content analysis was used to allow the themes to emerge from the quotes [9]. Interview tapes were listened to and transcripts read repeatedly to ensure accuracy and to include relevant information from field notes (e.g., pauses, laughter, overall mood of participants). The basic unit of analysis was defined as quotes from the transcription that represented participants’ experiences of PA. Quotes were clustered with other quotes with similar meaning, forming raw data themes [9]. Raw data themes were inspected for overlap and commonalities and, where appropriate, merged together to form higher-level themes. Higher level themes were labelled ‘first-order themes’ or ‘second-order themes’ accordingly with the highest level themes labelled ‘general dimensions’. General dimensions represented the highest-level, where no more themes could be uncovered [25].

A peer review process was used throughout the analysis to enhance reliability of the interview data. Two researchers (M.R and T.M) independently read the transcripts, identified quotes and clustered themes. Inter-rater reliability for the 11 items ranged from .60 to 1.00. The mean inter-rater reliability for these items was .78 (*SD* = 0.13). According to Landis and Koch [26], this result suggests that there is ‘substantial’ agreement’ between the two raters, and falls just short of being ‘almost perfect’ agreement (cutpoint = .80; p. 165). After discussions, the researchers reached consensus on assignment of the raw data and higher-level themes. Following procedures outlined by Scanlan et al. [24], when discrepancies existed in forming and naming the themes, the researchers re-examined the transcripts and conferred until all points of dispute were resolved. Inter-rater reliability was then equal to 1 as there was full agreement on all higher level themes. The two researchers who undertook the content analysis agreed that saturation in data was attained as no new themes emerged from the final two interviews.

The themes were classified under two dimensions: (i) barriers to, and (ii) facilitators of PA. A theme was categorised as a barrier if participants claimed that it hindered or prevented their participation in PA. Whereas a theme was categorised as a facilitator if participants believed that it encouraged their participation in PA. The number of ‘active’ and ‘inactive’ participants reporting each theme was recorded.

## Results

The two general dimensions of ‘barriers’ and ‘facilitators’ were made up of 11 second-order themes, which are presented in Table 2. In the following section, these themes are discussed in order of diminishing prevalence within each dimension, and the prevalence of the theme reported by the ‘active’ and ‘inactive’ groups stated.

**Table 2:** Barriers to and facilitators of physical activity for active and inactive participants

	**Active (n = 6)**	**Inactive (n = 9)**	**Total (n = 15)**
**Barriers**			
Low mood	3	5	8
Negative perceptions of health and life changes	0	7	7
Lack of motivation to exercise	0	6	6
Perceived external obstacles	2	4	6
Physical restrictions	1	4	5
Fear of exercise	2	3	5
Lack of knowledge regarding exercise	0	3	3
**Facilitators**			
Experiencing the psychological benefits of exercise	6	3	9
The positive role of others	4	4	8
Having a reason for exercising	4	2	6
Using psychological strategies	3	1	4

### Barriers to physical activity

#### Low mood

Eight participants (3 active (50%) and 5 inactive (56%)) stated that low mood was a major barrier to their PA. This is not surprising given that all participants in this study had been identified as having moderate depressive symptoms. The effects of low mood including feeling teary and emotional, and feelings of uselessness resulted in a permanent or temporary decrease in PA, and increase in more sedentary behaviours. One participant summarised this sentiment, saying “It’s not the physical side that stopped me from getting up and walking around the block, it’s more the mental side of things”. Another said “when I am feeling down, I can hardly even get myself out of bed, let alone do exercise”.

#### Negative perceptions of health and life changes

Seven participants (0 active (0%) and 7 inactive (78%)) stated that negative perceptions of their health and other life changes as a result of their heart disease was a barrier to being physically active. Coming to terms with getting older, having a sense of losing their health and dealing with the shock of developing the heart problem were difficult for some participants. One participant thought he was on the “downward slide” after having his heart attack and that it was “harder to exercise when you thought like this”. Others commented “it was a bit of a shock when I had this heart attack. I really thought I was bullet-proof. I realise I’m not, and this has slowed me down” and “up until the heart attack I felt quite fit, but since then I feel older and slower”. For some, the heart event brought unanticipated forced retirement, which exacerbated inactivity. One participant believed not working was “one of the biggest contributors to lower physical activity”. He said, “I used to be so active at work and now I don’t have that anymore”.

#### Lack of motivation to exercise

A lack of interest or desire to exercise was a common barrier reported by six participants (0 active (0%) and 6 inactive (67%)). Some acknowledged laziness or boredom with exercise as the problem. One commented, “I just don’t feel like it, I can’t be bothered.” Others, while aware of the benefits, could see no obvious reason or necessity to be physically active. One participant who was on the waiting list for bypass surgery described this lack of motivation: “I have no incentive at the moment to get fit, really nothing.” For others, if they couldn’t do their preferred type of exercise (due to, for example, injury or financial constraints), they found that “motivation gets harder all the time”.

#### Perceived external obstacles

Six participants (2 active (33%) and 4 inactive (44%)) identified external obstacles to PA. A concern for some participants was that the exercise they enjoyed was too expensive (e.g., playing golf or using a gym regularly). Comments such as “I can’t afford the petrol to get to the gym” and “I would love to play golf a few times a week, but just can’t afford it” highlight the financial barrier to being physically active. One participant described the financial constraints as being “the biggest killer” in stopping her from doing the exercise she wanted. Weather conditions, particularly cold and wet weather and reduced daylight hours in winter were also a deterrent for PA involvement. One participant commented “weather’s a big thing, if it’s cold and wet no one wants to go out and do exercise”.

#### Physical restrictions

Five participants (1 active (17%) and 4 inactive (44%)) commented that they felt restricted by their heart problem, particularly in terms of reduced energy levels, with one saying “you don’t have the same stamina and the same reserves within your body. Your energies are all totally depleted after heart surgery.” In addition, some participants had developed another physical problem (such as leg, knee or foot complaints) since their cardiac event that had become the major barrier preventing them from being physically active. For them, the inactivity was “frustrating”. One commented, “It’s been this vicious circle, that while doing exercise, I suffered the injury and it was painful. It was more painful than a heart attack”.

#### Fear of exercise

Five participants (2 active (33%) and 3 inactive (33%)) stated that a feeling that the exercise might “do them harm” restricted them from engaging in regular PA. Fears of “causing more damage to the heart” or damaging other body parts were a disincentive. One participant noted the “mental worry” caused by unfamiliar sensations and twinges. Another commented, “I’m not sure how this twisting and turning is going to affect me, so I’m a little bit cautious”.

#### Lack of knowledge regarding exercise

Three participants (0 active (0%) and 3 inactive (33%)) were uncertain about the benefits of exercise and the correct type, amount and intensity required for people with heart disease, which acted as a barrier. Participants described this as: “I just don’t know how much I can do now, especially when I get out of breath. But I am not sure I get anything from my walks if I don’t get out of breath” and “I can walk very slowly for a fair distance, but that’s not much good. You’ve got to put a bit of effort into it.” Similarly, another participant believed he needed to feel “rejuvenated” after the walk and because he didn’t, he wondered “what benefit can I get out of it”?

### Facilitators of physical activity

#### Experiencing the psychological benefits of exercise

Nine participants (6 active (100%) and 3 inactive (33%)) spoke about how experiencing the psychological benefits of exercise aided in maintaining PA. Participants commonly continued to exercise because it made them feel better psychologically, either by relaxing them, taking their mind off problems, such as pain or stresses, or enhancing their sense of achievement. One participant believed that exercise was “like a therapy for your mind and body” and once he was exercising his “mental attitude changed for the better.” Another participant spoke about how the exercise helped with his depression, stating that the exercise “makes you feel good within yourself and gives you a mental uplift”.

#### The positive role of others

Eight participants (4 active (67%) and 4 inactive (44%)) mentioned the importance of the partner, family and close friends in maintaining PA. Participants were highly motivated by support offered by significant others, either through encouragement to be active or by having an exercise companion. Participants commented “without my wife’s support to exercise, there is no way I would’ve got to the level I am now” and “my wife comes out 2 or 3 times per week, so she’s been very supportive that way”. Support given by the CR staff was also very important to many participants. The ability of staff to increase confidence, provide guidance and encouragement and to monitor participants’ health and progress during the CR program were important factors in participants’ maintenance of PA. One participant stated: “the time I spent at the [cardiac] rehab was fabulous because we had people there who were checking how we were feeling and our levels”. For some participants, exercise provided them with a chance to interact socially with other people. One participant said that through her exercise class “you get to see other people and you’re not by yourself all the time”. This encouraged her to maintain weekly class attendance. A few participants mentioned how important it was to have a positive role model for exercise: “I met this guy at rehab who had not exercised much in the past, but had started, little by little, and now exercises every day. I thought, “if he can do it, surely I can too”.

#### Having a reason for exercising

Having a reason for exercising was a facilitator reported by six participants (4 active (67%) and 2 inactive (22%)). A number of participants stated that doing the exercise for someone other than themselves was their major motivation for exercising. They spoke about having “someone worth fighting for” as being the driving force behind taking part in the necessary exercise to improve their health. For some, this person was a partner or family member: “I owe it to my partner. You’ve got to get better for your partner” and “I want to enjoy watching my grandsons grow up”. For other participants, the source of motivation was derived from a sense of obligation to health professionals: “I don’t want to let down those doctors who saved my life”. Another commonly reported reason for exercising was to improve health and live longer. One participant stated she exercised because she knew it was “good for her heart and body”. Other participants commented that they were “not ready to lay down and die” and that, by exercising “you’re helping yourself live a little longer”.

#### Using psychological strategies

Four participants (3 active (50%) and 1 inactive (11%)) reported psychological strategies, such as goal setting, positive self-talk, eliminating excuses, and prioritising exercise by writing it in a diary, as being facilitators for PA. With regard to setting goals, participants explained, “it’s important to always have goals in front of you”, and “having goals which are meaningful to you and the family are worthwhile….they give you a target”. One participant used self-talk to help her deal with the discomfort she sometimes felt while walking. She would say things like “come on, you can do this” and “you’ve only got one more to go”. For some participants, eliminating the excuses that encourage inactivity was helpful in ensuring they participated in adequate levels of physical activity. One participant stated emphatically “get rid of the excuses. I very quickly tore down those avenues that allowed me to not exercise so often”.

### Active versus inactive participants

Inactive participants tended to report more barriers (*M* = 3.22, *SD* = 1.72) than active participants (*M* = 1.50, *SD* = 0.55). In particular, negative perceptions of health and life changes, lack of motivation to exercise and lack of knowledge regarding exercise were not reported as barriers by any active participants. Conversely, facilitators were more frequently reported by active participants (*M* = 3.17, *SD* = 0.98) than by inactive participants (*M* = 1.00, *SD* = 0.87). In particular, inactive participants experienced few of the psychological benefits of PA. In comparison, at least half of the active participants identified each facilitator as being important to maintaining PA. These results offer support to our hypothesis that the salience of barriers and facilitators would differ in terms of the activity level of participants.

## Discussion

There is a paucity of data examining the PA experiences of people with CHD and depressive symptoms. The aim of the current study was to provide an in-depth account of barriers to and facilitators of PA in this population. The rich descriptive data derived from participants highlights the hindrances and motivators to maintaining PA after a cardiac event. There were numerous factors that negatively impacted on PA levels, including having negative perceptions towards health and life changes as a result of the cardiac event, having low mood and low motivation to exercise, feeling physically restricted or fearful of exercise, lacking knowledge regarding exercise and perceiving external barriers, such as financial constraints and weather conditions. These barriers were highly salient for a number of participants, particularly those who were inactive, and often appeared to have a negative influence on PA levels.

The results of the present study highlight the breadth of intrapersonal barriers. The intrapersonal barriers reported, such as lack of motivation, low mood, physical restrictions and negative perceptions of life changes, provided a major hindrance to participants’ ability to be physically active. Although there was a small number of participants in this study who reported financial concerns and weather conditions as being potential barriers, overall, environmental and organisational factors, such as lack of safety, access to facilities and lack of time were not mentioned as being problematic for the present sample. This finding concurs with those of Fleury et al. [13] who in their study of post-CR participants also reported that intrapersonal barriers to PA comprised the majority of overall barriers.

The dominance of intrapersonal factors as barriers to PA participation reported by participants with CHD and depressive symptoms may be a unique finding for people with a chronic illness, as numerous studies of the general population have identified a major influencing effect of environmental barriers on PA participation [27,28]. Surprisingly, not one participant in the current study reported that they did not have time to exercise. Perhaps this could be due to the fact that most of the participants were no longer working full-time, and had more time to invest into exercise than working people. Or maybe the intrapersonal barriers such as lack of motivation and negative perceptions of health changes were more salient to people with depression and CHD. It is also possible that, if these intrapersonal factors were addressed and overcome, a new set of barriers at environmental and institutional levels may be uncovered. Further investigation of this possibility is required.

Participants reported low mood to be a barrier to PA in its own right and in addition, low mood appears to be linked to a number of the other barriers. Lack of motivation, negative perceptions of health and life changes, fear and uncertainty are all intertwined with depression and low mood [15]. It is possible that, because the participants in the present study were also experiencing depressive symptoms, the ability to overcome barriers, such as lack of motivation and negative perceptions, may have been even more difficult than would be the case for people without depressive symptoms. It is likely that there is a confounding and complex relationship between depression, CHD and maintenance of PA. This hypothesis requires further investigation using quantitative methods.

In support of our hypothesis was the finding that barriers to PA were more frequently reported by inactive individuals than by active individuals. In particular, holding negative perceptions about life changes and health and having a lack of motivation to exercise were not considered a barrier to being physically active for any participants who were active, yet they were problematic to the majority of inactive participants. It is possible that simply being active provides motivation to be continually involved in PA and encourages positive perceptions about the changes to one’s life and health. Active individuals may be faced with the same barriers as inactive individuals, but have been able to overcome these barriers more effectively. Interestingly, low mood appeared to impact on PA levels of both active and inactive people. Health professionals can use this information to understand the possible barriers to PA, especially for people who are inactive, and to attempt to help patients overcome these barriers.

Participants in the current study also identified a number of factors that assisted in motivating them to engage in PA. Active participants reported more facilitators to PA than did inactive participants. In particular, being aware of psychological changes associated with exercise was an important facilitator in maintaining PA. This acted to inspire and encourage participants to adhere to their PA program. Other studies with cardiac and psychiatric populations have also identified mental health benefits to be a major motivator to PA [29,30]. It is possible that because the participants in the current study were experiencing depressive symptoms, they may have been even more aware of any positive psychological changes resulting from exercise. These possibilities could be the focus of further quantitative studies.

For many participants, especially those who were active, one of the key facilitators mentioned was the importance of having a reason for exercising. The present results indicate the importance of identifying the main reasons for exercising and then focussing on those reasons whilst exercising to ensure PA maintenance. Surprisingly, few qualitative studies in either cardiac or non-cardiac populations have reported this as a facilitator, suggesting that it might be particular to people with CHD and depression. One study reported CR participants who consistently engaged in exercise programs used their desire to survive and improve their health as the driving force in their motivation to exercise [31]. Encouraging people to identify the main reasons for exercising could be a very useful strategy in PA interventions, particularly for those with depression, for whom having a definite focus and engaging in goal setting can be important in maintaining PA [15]. Again, this possibility requires focussed research.

The present study provides an in-depth analysis of previously unexplored experiences of PA for people with depressive symptoms and CHD. Despite the strength of the findings in the current study, there were a number of limitations. First, the sample was not necessarily representative of the wider cardiac population. All participants had taken part in an earlier quantitative study undertaken by the same researchers and had expressed an interest in being involved in further research. In addition, the participants were all from an Australian or European background. Caution should therefore be exercised when generalising these results to CHD patients from other cultural backgrounds. Second, the CDS was administered up to four weeks prior to the interview, thus it is unclear whether depressive symptoms were present at the time of the interview. Importantly, previous studies have demonstrated that patients’ depression levels remain largely stable in the 12 months after a cardiac event [32]. Therefore, it is highly likely that participants were still experiencing at least some degree of depressive symptoms at the time of the interview.

The findings from the current study provide a preliminary depiction of some of the relevant barriers to and facilitators of PA for a group of participants experiencing both depressive symptoms and CHD. To further validate and verify these findings, a large scale quantitative study is required. A study of this type could include a sample of both depressed and non-depressed cardiac patients to ascertain the extent or prevalence of these barriers and compare the relative importance of the barriers to and facilitators of PA. Investigations could also include whether or not the barriers and facilitators were present before the cardiac event. In the current study, there were insufficient numbers to investigate whether there were differences in barriers and facilitators for males and females. A larger study is required to investigate gender differences in barriers and facilitators. The gender imbalance in this study is a result of the commonly seen larger number of males compared to females who experience a cardiac event (ratio 80:20).

The information gained through the current research may be useful in developing and implementing an intervention to encourage PA maintenance for people with CHD and depression. These findings could also provide valuable information for health care professionals working with both active and inactive people with CHD, particularly those in hospital- and community-based CR programs. Working with patients to identify and overcome relevant barriers and promote facilitators of PA could assist health professionals to be sensitive to the needs of people with CHD and depressive symptoms, and possibly improve health outcomes. The prevalence of depression in people with CHD, and the negative effect of the combination of CHD and depression on physical and psychological health together highlight the importance of the present research.

## Abbreviations

CHD: Coronary heart disease; PA: Physical activity; CR: Cardiac rehabilitation.

## Competing interests

The authors declare that they have no competing interests.

## Authors’ contributions

M.R conceived the study, carried out data collection, conducted data analyses and led the writing of the manuscript. B.M helped draft the manuscript. S.B participated in the design of the study and helped draft the manuscript. T.M conceived the study, participated in design of the study, assisted in data analyses and helped draft the manuscript. All authors read and approved the final manuscript.
